# SDMPH 10-year Anniversary Conference Modified Delphi Study

**DOI:** 10.1017/dmp.2024.251

**Published:** 2024-11-27

**Authors:** Eric S. Weinstein, Joseph Cuthbertson, Frederick Burkle, Hannah B. Wild, Rebekah Cole, Tehnaz Boyle, Jessica Ryder, Jeffrey Franc, Matthew Turek, Donal O’Mathuna, Wayne Cascio, Anja Westman, Manuela Verde, Marta Caviglia, David Eisenman, Emily Holbrook, Angus Jameson

**Affiliations:** 1 Department of Emergency Medicine, Morsani College of Medicine, University of South Florida, Tampa, FL, USA; 2 University of South Florida, Health Center for Advanced Medical Learning and Simulation (CAMLS), Tampa, FL, USA; 3 CRIMEDIM - Center for Research and Training in Disaster Medicine, Humanitarian Aid and Global Health, Università del Piemonte Orientale, Novara, Italy; 4 Monash University Disaster Resilience Initiative, Monash University, Monash, VIC, Australia; 5 Curtin University, Faculty of Health Sciences, Bentley, WA, Australia; 6 Global Scholar Woodrow Wilson International Center for Scholars, Washington, D.C., USA; 7 Program for Global and Rural Surgery University of Washington, Seattle, WA, USA; 8 Explosive Weapons Trauma Care Collective, International Blast Injury Research Network University of Southampton, Southampton, UK; 9 Uniformed Services University of the Health Sciences, Bethesda, MD, USA; 10 Boston Medical Center Department of Emergency Medicine, Boston, MA, USA; 11 University of Colorado Anschutz Medical Campus School of Medicine, Emergency Medicine; Team Rubicon; 12 Department of Emergency Medicine University of Alberta, Edmonton, Alberta Canada; 13 Defense Advanced Research Projects Agency, Arlington, VA, USA; 14 College of Nursing and the Center for Bioethics and Medical Humanities, The Ohio State University, Columbus, OH, USA; 15 Environmental Protection Agency, Washington, D.C., USA; 16 Örebro University Hospital, Emergency Department, Örebro, Sweden; 17 UCLA School of Medicine, Division of GIM/HSR, Los Angeles, CA, USA

**Keywords:** disaster medicine, education, public health professional, weapons, triage, mass casualty incidents

## Abstract

**Objectives:**

The SDMPH 10-year anniversary conference created an opportunity for a researcher to present at a professional association conference to advance their research by seeking consensus of statements using Delphi methodology.

**Methods:**

Conference attendees and SDMPH members who did not attend the conference were identified as Delphi experts. Experts rated their agreement of each statement on a 7- point linear numeric scale. Consensus amongst experts was defined as a standard deviation < = 1. Presenters submitted statements relevant to advancing their research to the authors to edit to fit Delphi statement formatting.

Statements attaining consensus were included in the final report after the first round. Those not attaining consensus moved to the second round in which experts were shown the mean response of the expert panel and their own response for opportunity to reconsider their rating for that round. If reconsideration attained consensus, these statements were included in the final report. This process repeated in a third and final round.

**Results:**

37 Experts agreed to participate in the first round; 35 completed the second round, and 34 completed the third round; 35 statements attained consensus; 3 statements did not attain consensus.

**Conclusions:**

A Delphi technique was used to establish expert consensus of statements submitted by the SDMPH conference presenters to guide their future education, research, and training.

Professional association conference proceedings summarize presentations discussing advances in treatment or technologies without the interaction between the presenter and audience. A professional association conference summary may be provided for attendees, those interested in the subject, or members of the professional association or society to add to their reference library. An extension of these summaries may outline future endeavors or pursuits by the organizing professional association or society. Consensus opinions via survey can be derived by activities at designated professional association conferences with the intent to issue specific statements to promote advances in clinical guidelines, treatment, or therapies.

The utilization of the scientific Delphi methodology at a conference of similarly educated and trained medical researchers and practitioners/responders to provide consensus statements as the sole objective of a professional association conference has proven to be a fruitful endeavor.^1^ These professional association conferences are designed based on the Delphi methodology, from the creation of the Delphi statements using an iterative process or discussion, to the conference participant in-person rounds of anonymous linear numeric responses of each statement until consensus or stability of answers is achieved. The *direct* output of these professional association conferences can be examined in the future to create clinical guidelines endorsed by conference attendees or the professional association or society that hosted or sent representatives to the conference.

The Society for Disaster Medicine and Public Health (Society) 10-year anniversary conference (SDMPH) took place on December 4^th^ and 5^th^ 2023 at the American Geophysical Union Conference Center in Washington D.C. The theme was “10 years on: Building the Discipline of Disaster Medicine and Public Health.” The sessions (Table 1) presented aspects of disaster risk reduction, disaster response, and the effects on vulnerable populations following these themes:

- Climate change and disaster response
- Conflict medicine and population health
- Advanced technology, artificial intelligence, decision aids, devices and equipment and their effect on mass casualty incident response
- Disaster medicine education, training, and competencies which are culturally and geographically relevant

The distinction between the prior professional association conferences designed specifically as a Delphi conference and the SDMPH is that the education and instruction opportunity permitted each presenter to learn the attendee’s interest or appreciation of the relevance of their research and to guide future direction of their research, education, training, and competencies through the scientific Delphi methodology *after* the conference.

## Methodology

The Delphi technique is a systematic process of forecasting using the collective opinion of panel members.^2^ In this study, the Delphi expert panel included in-person and virtual SDMPH participants, as well as Society members who did not attend the SDMPH. The structured method of developing consensus among panel members using Delphi methodology has gained acceptance in diverse fields of medicine.^3^ The Delphi method assumed a pivotal role in the last few decades to develop best practice guidance using collective intelligence where research is limited, ethically or logistically difficult, or evidence is conflicting.^4^ Delphi studies are modified by how the initial first round statements are created. This includes the use of open-ended questions to panel members; through an iterative process amongst authors, focus groups, or panel members; or other processes. Modified Delphi (mD) study statements were created by the SDMPH presenters and then an internal focus group of lead authors discussed and edited these statements to meet the format of Delphi statements for the Stat59 statistical analysis platform (Stat59 Services Limited, Alberta, Canada). The initial 32 statements submitted by presenters included statements from the Public Health Extreme Events Research (PHEER) network (Presentation # 3) and Telemedicine (Presentation # 9) (Table 1). These statements were excluded from consideration due to the Internal Focus Group determining that the total number of statements should ideally be less than 40 and both topic statements would attain consensus. In the end, statements were edited to fit the Delphi format leading to 38 statements to be presented to the Delphi Panel of SDMPH participants (Table 2).

SDMPH participants, in-person and virtual, were asked to participate as mD experts in a 3-round mD survey study seeking consensus of statements regarding research activities, training, education, and/or competencies related to topics presented at the SDMPH (*n* = 127). Also, Society members who were not attending were asked to participate in the Delphi (*n* = 230). Introductory emails were sent November 21, 2024 explaining the project objectives and the mD to these potential mD experts.

mD experts that agreed (*n* = 37, 25 SDMPH participants and 12 Society members not participating) were sent an email from the Stat59 mD organizational program with a link to the Stat59 website consent page. Each mD expert registered an account, validated it, and was sent a new email to log into their secure webpage to begin the first mD expert consensus round no later than 1359 GMT 4 December 2023, the start of the SDMPH. mD experts that had not logged into the system were asked to verify their access and log in and asked to notify the author if they had not received the introductory email, with instructions on how to ensure future emails were received.

Once the mD experts logged in, they were provided with a formal explanation of the mD methodology and informed consent was obtained. For informed consent (Supplemental Digital Content link to Stat59 Consent Page), participants were notified that they were anonymous volunteers who could withdraw at any time, that participation or withdrawal would not impact their employment, and that their data was secure (Supplemental Digital Content link to Stat59 Security Page). mD experts received a reminder email to complete this first round before the round closed.

The next page was the list of 38 statements with instructions to rate each statement on a 7-point linear numeric scale, where 1 = strongly disagree and 7 = strongly agree. With this initial set of statements, the mD expert was asked to answer 4 demographic questions. Consensus amongst mD experts was defined as a standard deviation ≤1.0. Statements that attained consensus after this first mD expert round were included in the final report.

Each mD expert received an email from the Stat59 program on December 9, 2023 notifying them that the second round was open; reminder emails were sent before the close of the second round (December 19, 2023) from the author that asked participants to log back into their Stat59 page that showed the mean response of all the mD experts for each statement that did not attain consensus and their own response for that specific remaining statement; they were then asked to reconsider their 7-point linear numeric scale for these remaining statements.

This process was repeated after the second mD expert round with statements that attained consensus included in the final report. The statements that did not attain consensus were advanced to the third and final round with the mD experts asked to reconsider these statements. Each mD expert received an email from the Stat59 program January 8, 2024 notifying them that the third and last round was open and reminder emails before the close of the third round January 18, 2024, similar to the above reminder emails. The third mD expert round produced statements that attained consensus to add to the first and second round consensus statements in the final report. Remaining statements after this third round were the final statements that did not attain consensus.

The University of South Florida Institutional Review Board (Tampa, Florida, USA) determined STUDY006415 met the criteria for exemption.

## Results

**Figure 1.**
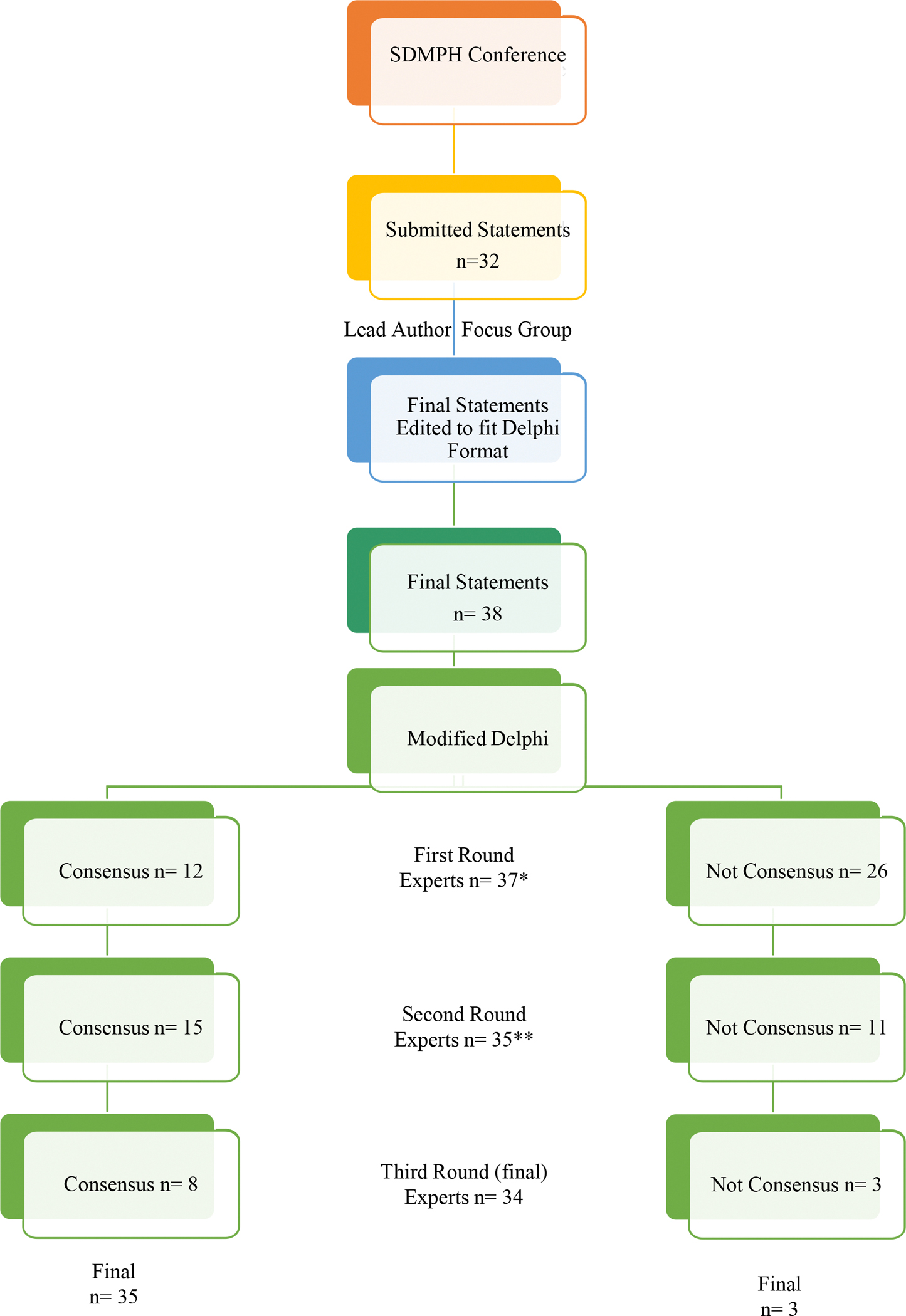
RESULTS * 25 SDMPH conference attendees, 12 who did not attend ** one participants dropped after 5 statements, another after 15 statements answered these statements were included in the analysis

As summarized in Figure 1, 37 mD experts (25 SDMPH participants and 12 Society members not participating) confirmed their participation, established a unique account on the Stat59, and completed the first mD expert round (Table 3). The majority of experts were from North America; worked at a university, or were involved in research, education, and training; were physicians; and had over 10 years of experience.

Twelve statements attained statistical significance with a standard deviation ≤1.0 after this first mD expert round, and achieved consensus (Table 4, first round, first section in bold). The 26 statements that did not attain statistical significance, with standard deviation >1.0, were advanced to the second mD expert round.

Thirty-five mD experts completed the second mD expert round, with 15 of the 26 statements that advanced to the second mD expert round achieving consensus (Table 4, second round middle section). The remaining 11 statements were unable to attain consensus and advanced to the third mD expert round.

Thirty-four mD experts completed the third and final mD expert round, 8 of the remaining 11 statements achieved consensus, with a total of 35 statements achieving consensus. (Table 4 third round, last section in bold). The remaining 3 statements were unable to attain consensus after 3 mD expert rounds and were not recommended for consideration for future research guidance (Table 5).

### Limitations

The PHEER and Telemedicine statements that were not included in the study impacted the overall number of statements but had no other bearing on the study.

The Delphi method seeks to arrive at group consensus by the aggregate of a panel of experts who rate a statement on a linear numeric scale. Franc et al. concluded that the sampling distribution tends to normality for sample sizes greater than or equal to 5. For Delphi studies using a 1–7 linear numeric scale, the use of the standard deviation for assessment of consensus and the mean for ranking is acceptable. Use of these parametric tests presents minimal risk of false consensus or of false ranking when compared to the non-parametric statistics of interquartile range and mean. This suggests that the linear 1–7 scale analyzed using the mean and standard deviation should become the preferred statistical method for Delphi studies.^5^

Study participants were skewed to North America and were physicians. The impact on the data cannot be determined. Certainly, having more input from non-physicians with less experience and from low-middle income countries is the goal of future mD studies to impact statements that could potentially attain consensus to guide future disaster medicine and public health preparedness education, research, and training.

The objective of the distribution of mD experts was to represent those involved with the education, training, and competencies of those who seek disaster risk reduction, disaster medicine response, or recovery as evidenced by their participation in the SDMPH or Society. The distribution of these mD experts favor those who had the means to attend the SDMPH or join the SDMPH, potentially impacting the study lacking the input from those from a resource-limited setting who could not attend or join.

An essential component of the Central Limit Theorem is that the average of sample means will be the population mean, or if 1 finds the average of all of the standard deviations in the sample, then 1 will find the actual standard deviation for the population.^6^ This will hold true regardless of whether the source population is normal or skewed, provided the sample size is sufficiently large (usually ≥30, the number of mD experts in this study per round averaged 29.67).^7^ The application of the Central Limit Theorem to this study infers that the 35 statements that attained consensus can be recommended to guide presenters with their current and future research.

## Discussion

The theme of the SDMPH “Building the discipline of Disaster Medicine and Public Health” is to retain the lessons learned after an exercise or an actual incident for the participating agencies or organizations to improve their unique education, training, competencies and response. The first day of the SDMPH focused on climate change and conflict in disaster medicine. The second day featured presentations on ethics and artificial intelligence, devices and equipment, equipment and training.

Short-term and long-term exposure to ambient air particle pollution cause a myriad of health and ecological effects (statements #1, 2).^8,9^ In particular, short-term exposures lasting hours to a few weeks and long-term exposures lasting months to years adversely affect cardiopulmonary health as evidenced by increased premature mortality, and hospitalization for cardiovascular disease (#1, 2).^10,11^ Individuals with prevalent heart disease, namely ischemic heart disease and heart failure, are at higher risk for having adverse health effects after exposure (#2).^12^ Wildland fire smoke is an important and growing source of particulate air pollution and is increasing overall average concentrations of fine particulate matter in most of the continental U.S. or at least arresting the overall improvement of air quality that has occurred over the next several years (#3).^13^ Yet, few health care professionals educate their patients to modify their exposure risk highlighting the potential benefit of education (#4, 5).^14,15^ When questioned, the attendees showed strong agreement that the education of health care professionals should include training on the care of people affected by wildfire smoke and that health care systems should increase the care for patients affected by wildfire smoke (#17, 18). Similarly, study participants agreed that a transdisciplinary approach is required to anticipate and mitigate the impact of emerging risks of floods on public health systems due to compromised treatment and care for people with noncommunicable diseases (#9, 23).

Humanitarian surgical care (HSC) in 21st-century armed conflict poses a complex nexus of challenges related to factors including shifting conflict dynamics, security constraints, and proliferation of unconventional actors within the humanitarian space.^16^

Study participants agreed that a focused approach is required to improve the quality of surgical care delivered to civilians in asymmetric conflict with forward surgical services with intact echelons of care to reduce mortality and morbidity (#19). The study participants agreed that HSC and the trauma stabilization point (TSP) should be considered to manage the full range of population needs including not only trauma-related but also non-traumatic emergencies (e.g., cesarean section), acute medical conditions, and uncomplicated minor injuries in addition to asymmetric warfare trauma (#12, 20, 24, 25).^17^

Study participants agreed that coordination between military and other armed groups must be developed based on the humanitarian trauma system needs of non-combatant citizens, military, and other armed groups (#26). At present, surgical training in high-resource settings is not well-suited for this breadth.^18^ Study participants agreed that capacity and capability of the humanitarian trauma system should be based on available expertise of humanitarian responders and the local health care delivery system (#27). Standardized pre-deployment training and competency assessment in core HSC procedures may contribute to ensuring quality of care. To adequately benchmark care quality, it is necessary to establish a minimum dataset (MDS) with legitimate-use sharing agreements (#21).^19^ Development of risk-adjusted perioperative mortality calculators for resource-constrained environments may support this process.^20^ Due to the high-threat environments in which HSC often occurs, there has been increased engagement from non-traditional actors.^21^ Establishing transparent credentialing and training in international humanitarian law (IHL) among all actors providing HSC in conflict may help ensure protection of humanitarian principles (#22).

Over the last 10 years ethical challenges presented and the rise of artificial intelligence provided disaster medicine researchers fertile subject matter to pursue. Three statements related to ethical issues with artificial intelligence (AI) in disasters attained consensus. Statement #34 noted that the ethical issues with AI for mass casualty incidents have not been thoroughly analyzed as examples are growing of specific situations where AI has been deployed during disasters without due consideration of its ethical considerations. These include cases where concerns about privacy and confidentiality of data occurred.^22^ Another concern relates to the quality of datasets used to train AI.^23,24^

Statement #11 about biases in datasets raising serious ethical concerns attained consensus and affirmed the importance of research into this issue. Given that many disasters occur in low-income countries, issues of justice need research related to AI. Nature carried a headline accompanying the release of a 2024 World Health Organization (WHO) report into the ethics of AI stating, “Medical AI could be ‘dangerous’ for poorer nations.”^25^ Statement #33 attained consensus, agreeing that AI may not benefit those impacted by disasters in low-income countries as much as those in higher-income countries. Further research is needed to examine and mitigate such disparities.

Many other ethical issues remain under-investigated with analysis urgently needed to avoid undermining AI’s potential benefits. With regards to the need for encryption of patient identifiers in AI tools, the study participants attained consensus with strong support (mean 6.6, SD 0.8) (#2). The study participants showed moderate support (mean 5.5, SD 0.9) for the need for strict statistical validation of AI tools prior to implementation (#29). In contrast, study participants showed weak support (mean 3, SD 1) for the replacement of traditional printed disaster plans with AI guided just in time interfaces (#30). Though each statement attained consensus, additional studies in concert with developing ethical solutions should foster more dedicated research of AI models.

Defense Advanced Research Projects Agency’s (DARPA’s) In the Moment (ITM) program will leverage insights from the statements that did [#6] and did not attain consensus (Not #1, 2). ITM research will assess how human decision-makers’ perceptions of an AI algorithm’s competence, value alignment and/or other factors might make a human decision-maker more likely to delegate a decision to the AI algorithm. ITM will also assess how the decision to delegate may vary depending on environmental conditions and/or personal context. DARPA shared insights from the study with the program’s performers. The insights will also inform the program’s Ethical, Legal, and Societal Implications (ELSI) processes through panel discussions at ITM workshops. The workshops are part of DARPA’s outreach to policymakers and practitioners.

Development, creation, and subsequent implementation of devices in the disaster medicine setting impose additional layers of ethical and practical considerations. The value of point-of-care ultrasound technology in the prehospital and resource-scarce environment is evident (#32) but finding ways to share this technology and knowledge through effective training programs has challenges that include sustainability and quality assurance once the training programs have concluded. One potential feasible solution to this challenge is the use of virtual meeting platforms and remote image review as quality assurance measures once the technology is implemented in a community (#16). These measures provide a means to also gauge impact of training programs and should be incorporated into training programs during the early planning stages in coordination with the host communities as standard practice. With the implementation of programs, sustainability should be an important consideration, but consensus was not attained on whether sustainability should impact the decision to implement a point-of-care ultrasound training program in specific communities (Not #3). This may be partially due to the ethical dilemma of withholding lifesaving technology that could impact mortality from a community during a time of need, such as a sudden-onset disaster or conflict.

Historically, after action reports (AARs) of exercises and incidents with long scene or transport times have identified gaps determining which patients require lifesaving interventions (LSI) and priority transport with the demand of patients exceeding the supply of monitoring devices. As the discipline of disaster medicine has grown over the last 10 years, government agencies cognizant of these AARs have dedicated research to address these gaps. Study participants support the design and creation of patient monitoring devices utilized in an MCI to enhance LSI and priority transport (#3, 4). Scene safety, ingress and egress routes, the placement of casualty collection points, advanced medical posts, transportation staging, incident command, and identification of patients in the continuous triage process of an MCI can be assisted by drones (#31). Data collected and stored by patient monitoring devices can be analyzed retrospectively to identify trends to improve prehospital processes during MCI responses (#5).

The NIGHTINGALE research and innovation project funded by the European Union’s Horizon 2020 program convened experts in technology, research, medical practitioners, and leading organizations in Europe that specialize in MCI response. The objectives include to: (1) upgrade evaluation of injured and affected populations (triage) using digital identification, traceability, fast diagnosis, continuous monitoring, and accurate classification of medical conditions; (2) optimize pre-hospital LSI and enhance utilization of assets, resources and capacities using AI-based solutions; and (3) enable shared response across emergency services and communication between emergency teams and with patients by developing augmented reality tools for first responders. This is consistent with the statement that prehospital MCI response training should be delivered to all first responders (#28).

High-fidelity simulation MCI response training will lead to evidenced-based practices for education and training of both civilian and conflict responders to enhance trainees’ stress tolerance and will accelerate trainees’ professional identity development (#8, 14, 15). As the discipline of disaster medicine has advanced, the recognition that responders are able to focus their attention through practices such as mindfulness breathing has led to research with military medical students investigating whether mindfulness-based practice can improve performance while under stress.^26^ The Sendai Framework calls for disaster medicine education to be incorporated into standard health professional medical curriculums consistent with the study participants’ acknowledgement that high-fidelity MCI response simulation training increases readiness for deployment (#7, 13).^27^ In addition, future studies should measure MCI response readiness outcomes during simulation within both civilian and conflict settings.

Scenario based simulation for MCIs and HCRs should become more accessible along with training on the use of rapid systematic assessment tools. Together these would maximize health care readiness for deployment to help ensure dynamic data-driven decisions are made with high confidence levels. A tool that could provide the template to support such a capability is the United Nations Public Health System Resilience Scorecard (Scorecard) (#10). The Scorecard is versatile, scalable, and could be easily modified. It is well suited to gather insights systematically and rapidly on needs and priorities using a consensus-based approach to determine which priority actions and needs from earlier planning should be kept or removed prior to deployment. The Scorecard process is designed to ensure a multidisciplinary team discussion and systemwide exploration of healthcare mission readiness, providing a high level of confidence of what is needed for success.

## Conclusion

This study demonstrates how an mD conducted at a professional association conference with the objective to provide a forum for presenters conducting research in disaster risk reduction, disaster medicine response, and recovery can provide consensus statements to guide the presenter’s current and future research to advance education, training, and competencies.

## Supplementary Material

Supplemental material

**Supplementary material.** The supplementary material for this article can be found at http://doi.org/10.1017/dmp.2024.251.

## Figures and Tables

**Table 1.** SDMPH Conference Presentations

Presentation	Title	Presenter
		
Keynote	NDMS: The Next 10 Years	Helga Marie Scharf-Bell, DNP, MSN, FNP-BC, RN, NDHP-BC
1	Assessing and Increasing Health System Resilience in Non-traditional Flood Areas	Benjamin J. Ryan PhD, MPH
2	Human Health Impacts of Wildland Fire	Wayne E. Cascio, MD, FACC
3	Climate Change and Community Resilience: the PHEER Network	David P. Eisenman, MD, MSHS
4	How Conflict Zone Trauma Stabilization Points Translate to the Resource Constrained MCI Response	Flavio Salio, PhD
5	Prehospital Point-of-Care Ultrasound to Guide Life Saving Damage Control Interventions and Priority Transport in Resource Scarce Environments	Jessica Ryder, MBBS, PhD
6	Humanitarian Surgery in 21st-Century Armed Conflict	Hannah B. Wild, MD
7	Artificial Intelligence Chatbots in Disaster Medicine: What is ChatGPT and How Do I Use It?	Jeffrey Franc, MD, FCFP.EM., MSc(Disaster Med), MS (Statistics), Dip Sport Med
8	DARPA’s In the Moment (ITM) Program: Human-aligned Algorithms for Making Difficult Battlefield Triage Decisions	Matt Turek, PhD
9	The Path of Telemedicine in Disaster Response	Tehnaz P. Boyle, MD, PhD
10	Ethics of Artificial Intelligence in Disaster Medicine	Dónal O'Mathúna, B.Sc.(Pharm), MA, PhD
11	Developing Wearable (Wristband) and Unmanned Aerial Vehicle (Drone) Advanced Technologies in Mass Casualty Incident (MCI) Response: The NIGHTINGALE PROJECT	Marta Caviglia, MD PhD
12	Augmented Reality in Education	Rebekah Cole, PhD, MEd
Keynote	Essential Disaster Medicine Education, Training and Competencies	Luca Ragazzoni, MD, PhD

**Table 2.** DELPHI STATEMENT REVIEW PROCESS

1 draft statement
**Coffee should have milk and sugar.**
The expert has 2 variables to decide on the 7-point linear numeric scale. The expert will have difficulty reconciling 2 linear numeric scales for each variable to arrive at one linear numeric scale answer for the statement.
Statement edited to 2 final statements
**Coffee should have milk.**
**Coffee should have sugar.**
Now the expert has 1 variable to decide on the 7-point linear numeric scale.

**Table 3.** Modified Delphi Expert panel demographics

Location of primary Mass Casualty Incident Response education, training, planning or operations employment (n= 35, 2 experts did not complete the demographic survey, select 1)	
Sub-Saharan Africa	2
East Asia and Pacific	0
Europe and Central Asia	5
Middle East and North Africa	2
Latin America and Caribbean	0
North America	25
**Primary employment (n=, select 1)**	
University or research center	27
Governmental Organization	6
Non-Governmental Organization	0
Private sector	0
Other	0
**Current profession (select all that apply)**	
Education/Training	24
Research	7
Response/Field operations	8
Physician	16
Nurse	6
EMT or paramedic	4
Physician Assistant	1
Public Health	1
Dentist	2
Administration and support	4
Simulation coder, designer, creator	4
Policy	1
Student: Physical Therapy Assistant	1
Biosecurity/Medical Intelligence	1
Operations and Systems	1
**Years of expertise in this field (n=35, select 1)**	
<5	4
6–10	6
11–15	11
>16	14
**SDMPH Member (n=35, select 1)**	
Yes	22
No	13
**Did you attend the SDMPH Conference? (n= 35, select 1)**	
In-person	12
Virtually	14
No	9

**TABLE 4.** STATEMENTS ATTAINING CONSENSUS

#	Round	Statement	n	mean	sd
1	1	Access to mass casualty incident response education can be enhanced with Scenario-Based e- Simulation (SBES).	37	6.2	0.9
2	1	Unencrypted data containing patient identifiers should never be transmitted to commercial artificial intelligence chat programs.	36	6.6	0.8
3	1	Patient monitoring devices allowing continuous monitoring of vital signs during mass casualty incidence responses can enhance lifesaving interventions.	36	5.9	1
4	1	Patient monitoring devices allowing continuous monitoring of vital signs during mass casualty incidence responses can enhance priority transportation decisions.	36	5.9	1
5	1	Data collected and stored by patient monitoring devices can be analyzed retrospectively to identify trends to improve prehospital processes during mass casualty incident responses.	36	6.4	0.9
6	1	Users will not trust artificial intelligence to make life or death mass casualty incident response triage decisions unless there is demonstrated alignment between artificial intelligence values and the users’ values.	36	6.1	1
7	1	High fidelity mass casualty incident response simulation training increases readiness for deployment.	36	6.5	0.8
8	1	High fidelity mass casualty incident response simulation training accelerates trainees' professional identity development.	35	5.9	1
9	1	A transdisciplinary approach is required to anticipate and mitigate the impace of flooding on health care delivery systems in areas not accustomed to flooding.	35	6.2	1
10	1	The United Nations Disaster Risk Reduction (UNDRR) public health system resilience scorecard is a method that can be used to guide disaster resilience and recovery actions in multiple communities and contries.	35	5.1	1
11	1	Potential biases in datasets used to train artificial intelligence algorithms generate serious ethical concerns.	35	5.9	0.9
12	1	The utility of the trauma stabilization point should be considered in all conflict settings even where fighting is sporadic or frontlines are poorly defined.	35	5.1	1
**13**	**2**	Disaster medicine education should be incorporated in standard health professional medical curriculums to support the Sendai Framework that advocates for training in disaster medicine.	35	6.5	0.8
**14**	**2**	Educational curricula in disaster medicine should be evidence based.	35	6.3	0.8
**15**	**2**	High fidelity mass casualty incident response simulation training enhances trainees' stress tolerance.	35	5.9	0.8
**16**	**2**	Virtual image reviews and regularly scheduled virtual meetings are sufficient for quality assurance after initiation of a mass casualty incident responst point-of-care ultrasound training program.	35	3.3	1
**17**	**2**	Health profession training programs should increase training and education to advance the care of patients affected by wildfire smoke.	35	5.5	1
**18**	**2**	Health care systems should increase response capacities to care for patients affected by wildfire smoke due to impacts of climate change, heat and wildfire.	35	5.8	1
**19**	**2**	In a humanitarian setting far-forward surgical services with intact echelons of care are necessary to reduce mortality and morbidity.	35	4.8	1
**20**	**2**	Humanitarian surgical care in conflict zones should encompass both trauma and non-trauma related surgical emergencies.	35	5.9	1
**21**	**2**	Humanitarian surgical care in conflict zones process and outcomes data should be recorded using agreed criteria to create a minimum dataset.	35	6	0.8
**22**	**2**	All actors providing humanitarian surgical care in conflict should be required to undergo externally verified training in International Humanitarian Law (IHL) and humanitarian principles.	35	6.4	0.7
**23**	**2**	The greatest impact of floods on health care delivery systems relates to compromised treatment and disruption of care for people with chronic illnesses.	35	5.5	0.9
**24**	**2**	The trauma stabalization point must be prepared to initially manage acute medical conditions in addition to trauma.	35	5.8	0.8
**25**	**2**	Penetrating and blast injuries should always be considered for referral to higher levl of care in the conflict setting.	35	5.9	1
**26**	**2**	Coordination between military and other armed groups must be developed based on the humanitarian trauma system needs of non-combatant citizens, military and other armed groups.	35	5.9	0.9
**27**	**2**	Capacity and capability of the humanitarian trauma system should be based on available expertise of humanitarian responders and the local health care delivery system.	35	5.8	1
28	3	Prehospital mass casualty incident resonse training shuld be delivered to all first responders.	34	6.8	0.5
29	3	First response agencies should require strict statistical validation of all artificial intelligence tools before implementation.	34	5.5	0.9
30	3	Artificial intelligence guided just-in-time interfaces should replace traditional printed disaster plans.	34	3	1
31	3	Drones can effectively assist triage procedures during mass casualty incident responses by integrating vital sensing capabilities.	34	5.6	0.9
32	3	Point-of-care ultrasound technology should always be available for use in prehospital mass casualty incident response.	34	4.7	0.9
33	3	Artificial intelligence algorithms may not have similar benefits for disaster victims in both high-income and low-income countries.	34	5.1	1
34	3	The ethical issues with artificial algorithms tor mass casualty incident response have not been thoroughly analyzed.	34	5.8	0.8
35	3	Uncomplicated minor injuries can be managed at the trauma stabiliation point	34	5.3	0.9

**TABLE 5.** STATEMENTS NOT ATTAINING CONSENSUS

#	Round	Statement	n	mean	sd
1	3	Artificial intelligence triage algorithms should prioritize human values during decision making.	34	5	1.3
2	3	Artificial intelligence mass casualty incident response triage algorithms that are aligned to human values could be fully automated to make treatment decisions.	34	3.5	1.2
3	3	A mass casualty incident response point-of-care ultrasound training program should only by offered if sustainable in the community.	34	4.9	1.3
