# Supplementary material for: SDMPH 10-year Anniversary Conference Modified Delphi Study
Source: Disaster Med Public Health Prep. Author manuscript; Available in PMC 2025 Dec 26. (PMC12741868; doi:10.1017/dmp.2024.251)
Supplement: Supplemental material [file NIHMS2127881-supplement-Supplemental_material.docx]

Supplemental Digital Content Links

Stat59 Security Page

<https://www.stat59.com/about/security>. Accessed 6 June 2024

SDMPH Conference Proceedings Modified Delphi Study Consent

<https://www.stat59.com/projects/delphi-consent-view?pid=503>. Accessed 6 June 2024
